# Primary caregivers of individuals with developmental disabilities or disorders in Canada: highlights from the 2018 General Social Survey – Caregiving and Care Receiving

**DOI:** 10.24095/hpcdp.45.5.04

**Published:** 2025-05

**Authors:** Sarah Palmeter, Siobhan O’Donnell, Sienna Smith

**Affiliations:** 1 Centre for Surveillance and Applied Research, Health Promotion and Chronic Disease Prevention Branch, Public Health Agency of Canada, Ottawa, Ontario, Canada

**Keywords:** developmental disorders, developmental disabilities, General Social Survey, caregivers, neurodevelopmental disorders, population surveillance, surveys and questionnaires

## Abstract

Using data from the 2018 General Social Survey – Caregiving and Care Receiving, we examined the characteristics of caregivers of people with developmental disabilities or disorders (DD) and the impacts of caregiving on these caregivers. The proportion of DD caregivers with optimal general and mental health was smaller than the proportion of non-caregivers. About two-thirds of DD caregivers reported feeling worried or anxious, or tired and almost half reported unmet support needs. However, compared with caregivers of individuals with other conditions, a significantly higher proportion of DD caregivers described their caregiving experiences as rewarding.

**Highlights**

- Characteristics of caregivers of individuals with developmental disabilities or disorders (“DD caregivers”) were compared with those of caregivers of individuals with other conditions and with those of non-caregivers.
- A smaller proportion of DD caregivers than non-caregivers reported optimal general and mental health.
- Many DD caregivers reported feeling worried or anxious, feeling tired and spending less time taking care of themselves due to their caregiving responsibilities.
- Almost half of DD caregivers reported unmet support needs, particularly financial support, government assistance or tax credits, occasional relief or respite care, and home care or support.
- Despite these challenges, a significantly higher proportion of DD caregivers described their caregiving experiences as rewarding or very rewarding compared with other caregivers.

## Introduction

Unpaid caregiving is increasingly common in Canada, driven by an aging population, the rising prevalence of disabilities and a growing emphasis on community- and home-based care.^1^,^2^ In 2018, about one in four people aged 15 years and older provided unpaid care to a friend or family member with a long-term health condition, disability or age-related issue in Canada.^3^

While all caregivers face unique challenges, those supporting individuals with developmental disabilities or disorders (DD) have distinct experiences. These caregivers (referred to as “DD caregivers”) often provide ongoing support that evolves throughout the care receiver’s lifespan.^4^,^5^ Their roles are important and wide-ranging, impacting the lives of children, youth and adults with DD.^4^,^6^,^7^

DD encompass a group of conditions characterized by differences in physical development, learning, language or behaviour, which can affect daily functioning.^8^ DD become apparent early in life and last throughout a person’s life. Common examples include intellectual disabilities, autism, attention-deficit/hyperactivity disorder, fetal alcohol spectrum disorder, Down syndrome and cerebral palsy.

Population-based studies in Canada have found that caregivers of children with DD report more health problems and poorer mental health than those caring for children without DD.^9^,^10^ Population-based studies from other countries also report poorer health outcomes, mental health challenges, increased financial struggles and lower well-being among DD caregivers.^5^,^11^

To better understand these aspects, we used data from the 2018 General Social Survey (GSS) – Caregiving and Care Receiving^12^ to examine the characteristics of caregivers of children, youth and adults with DD living in Canada and to describe the impacts of caregiving on caregivers.

## Methods

***Data source and study population***

The GSS – Caregiving and Care Receiving is a national survey of people aged 15 years and older living in Canada’s 10 provinces.^12^ The 2018 survey collected information on primary caregivers, that is, people who provided help or care to family members, friends or neighbours with a long-term health condition, physical or mental disability or aging-related problem in the past 12 months. Paid help or help provided on behalf of an organization were not within the scope of this survey.

Our study focus was on primary caregivers of people whose “main health condition or problem for which they have received help” was a “developmental disability or disorder.”^12^

The full unweighted sample of the 2018 GSS was 20 258, of whom 248 self-identified as DD caregivers, 7416 as caregivers of individuals with conditions other than DD (referred to as “other caregivers”) and 12 594 as non-caregivers. About 1% of caregiver interviews and 2% of non-caregiver interviews were conducted by proxy when the respondent did not speak English or French or could not participate in the survey for health reasons.^12^

***Statistical analysis***

Descriptive analyses were conducted to examine DD caregivers’ and care receivers’ sociodemographic characteristics, the type of care provided, caregivers’ health status, the impacts of caregiving on caregivers, caregiver supports and unmet support needs.

All estimates were weighted to be representative of all non-institutionalized persons aged 15 years and older, living in the 10 provinces of Canada, using sample weights provided by Statistics Canada for this survey.^12^ Bootstrap methods were used to calculate variance estimates, including 95% confidence intervals (CI) and coefficients of variation. The estimates for DD caregivers were compared with estimates for other caregivers or for GSS respondents who were not caregivers, where appropriate. The associated 95% CIs were also compared and non-overlapping 95% CIs were considered statistically significantly different. 

Analyses were carried out using statistical package SAS Enterprise Guide version 8.1 (SAS Institute Inc., Cary, NC, US).

## Results

Based on data from the 2018 GSS, 4.5% (95% CI: 3.6%–5.3%) of caregivers provided care to a family member or friend with DD. DD were ranked as the seventh most common condition cared for, while aging or frailty (22.7%; 21.2%–24.3%), cancer (9.9%; 8.8%–10.9%) and mental illness (9.7%; 8.5%–11.0%) were the three most common in the survey (data not shown).

***Sociodemographic characteristics***

The mean age of DD caregivers at the time of the survey was 45.7 years, and 58.9% were female. About one-fifth identified as a visible minority, 55.7% had a postsecondary education and 59.1% were employed ( Table 1).

**Table 1:** Sociodemographic characteristics, care provided, health status, impacts of caregiving, supports and unmet support needs of DD caregivers,a other caregiversb and non-caregivers,c Canada (excluding territories), 2018

Variable	Aidants de personnes avec des ITD^ a^ % (IC 95 %)	Autres aidants^ b^ % (IC 95 %)	Non-aidants^ c^ % (IC 95 %)
Caractristiques sociodmographiques			
**ge moyen, en annes**	45,7 (42,3 49,2)	49,2 (48,4 49,9)	46,1 (45,9 46,3)
Sexe			
Fminin	58,9 (48,6 69,2)	53,8 (52,0 55,5)	49,5 (48,9 50,1)
Masculin	41,1 (30,8 51,4)	46,2 (44,5 48,0)	50,5 (49,9 51,1)
Origine ethnique^ d^			
Appartient une minorit visible	20,8 (12,3 29,2)^ E^	16,7 (15,0 18,4)	25,0 (23,8 26,1)
N’appartient pas une minorit visible	79,2 (70,8 87,7)	83,3 (81,6 85,0)	75,0 (73,9 76,2)
Plus haut niveau de scolarit			
tudes secondaires ou moins	44,3 (33,8 54,9)	36,6 (34,7 38,5)	42,2 (41,0 43,3)
tudes postsecondaires	55,7 (45,1 66,2)	63,4 (61,5 65,3)	57,8 (56,7 59,0)
Emploi			
Employ (a travaill ou s’est absent d’un emploi au cours de la semaine prcdente)	59,1 (48,6 69,6)	61,2 (59,5 63,0)	61,3 (60,2 62,4)
Chmeur (n’avait pas d’emploi au cours de la semaine prcdente)	40,9 (30,4 51,4)	38,8 (37,0 40,5)	38,7 (37,6 39,8)
**ge moyen du bnficiaire de soins, en annes**	22,5 (19,5 25,4)	68,9 (68,0 69,8)	S.O.
Sexe du bnficiaire de soins			
Fminin	35,8 (26,0 45,6)	63,3 (61,5 65,1)	S.O.
Masculin	64,2 (54,4 74,0)	36,7 (34,9 38,5)	S.O.
Relation entre le bnficiaire de soins et l’aidant			
Enfant de l’aidant	62,2 (52,8 71,7)	5,5 (4,7 6,2)	S.O.
Frre ou sœur de l’aidant	20,8 (11,5 30,0)^ E^	4,7 (3,9 5,5)	S.O.
Petit-fils ou petite-fille de l’aidant	5,6 (2,4 8,8)^ E^	^ F^	S.O.
Autre^ e^	11,4 (6,4 16,4)^ E^	89,2 (88,1 90,4)	S.O.
Situation de vie			
L’aidant fait partie du mme mnage que le bnficiaire de soins	79,2 (72,3 86,1)	33,6 (31,7 35,5)	S.O.
L’aidant et le bnficiaire de soins font partie de mnages diffrents	20,8 (13,9 27,7)^ E^	66,4 (64,5 68,3)	S.O.
Soins fournis			
Le bnficiaire de soins a au moins un autre aidant (rmunr ou non)			
Oui	83,9 (76,3 91,6)	71,1 (69,3 73,0)	S.O.
Non ou ne sait pas	16,1 (8,4 23,7)^ E^	28,9 (27,0 30,7)	S.O.
**Nombre moyen d’heures de soins par semaine**	29,1 (22,7 35,5)	13,4 (12,5 14,2)	S.O.
Aide pour le transport			
Oui	86,2 (79,1 93,3)	72,7 (70,9 74,4)	S.O.
Non	13,8 (6,7 20,9)^ E^	27,3 (25,6 29,1)	S.O.
Aide pour la prparation des repas, la vaisselle, le mnage, la lessive ou la couture			
Oui	80,0 (72,4 87,5)	55,3 (53,5 57,1)	S.O.
Non	20,0 (12,5 27,6)^ E^	44,7 (42,9 46,5)	S.O.
Aide pour l'organisation et la planification des soins			
Oui	60,5 (50,2 70,8)	39,9 (38,2 41,7)	S.O.
Non	39,5 (29,2 49,8)	60,1 (58,3 61,8)	S.O.
Aide pour les soins personnels			
Oui	58,8 (49,0 68,6)	27,7 (26,2 29,3)	S.O.
Non	41,2 (31,4 51,0)	72,3 (70,7 73,8)	S.O.
Aide pour la gestion des finances			
Oui	44,5 (34,9 54,2)	32,0 (30,3 33,7)	S.O.
Non	55,5 (45,8 65,1)	68,0 (66,3 69,7)	S.O.
Aide pour les procdures ou traitements mdicaux			
Oui	37,5 (28,5 46,4)	26,0 (24,5 27,5)	S.O.
Non	62,5 (53,6 71,5)	74,0 (72,5 75,5)	S.O.
tat de sant			
Sant gnrale			
Excellente/trs bonne	40,5 (30,7 50,4)	48,5 (46,6 50,3)	58,6 (57,3 59,9)
Bonne	41,9 (32,0 51,8)	34,4 (32,7 36,1)	30,1 (28,9 31,3)
Passable/mauvaise	17,5 (11,1 24,0)^ E^	17,1 (15,8 18,5)	11,3 (10,6 12,1)
Sant mentale			
Excellente/trs bonne	45,7 (36,1 55,3)	51,9 (50,1 53,7)	64,1 (62,7 65,4)
Bonne	32,8 (24,1 41,5)	32,5 (30,8 34,2)	26,5 (25,3 27,7)
Passable/mauvaise	21,5 (13,0 29,9)^ E^	15,6 (14,2 17,0)	9,4 (8,6 10,2)
Stress			
Plupart des journes pas du tout stressantes/pas trs stressantes	25,8 (16,1 35,5)^ E^	28,1 (26,5 29,6)	37,4 (36,2 38,6)
Plupart des journes un peu stressantes	46,9 (37,2 56,5)	47,2 (45,4 49,0)	42,4 (41,2 43,6)
Plupart des journes assez ou extrmement stressantes	27,3 (19,4 35,2)	24,7 (23,2 26,3)	20,1 (19,1 21,2)
Satisfaction l’gard de la vie^ f^			
Satisfait/trs satisfait	77,2 (69,9 84,5)	80,5 (79,0 82,0)	86,9 (86,0 87,8)
Ni satisfait, ni insatisfait	15,0 (8,4 21,7)^ E^	10,2 (9,0 11,3)	7,2 (6,6 7,9)
Trs insatisfait/insatisfait	7,8 (3,7 11,8)^ E^	9,3 (8,2 10,5)	5,9 (5,2 6,5)
Bonheur^ f^			
Heureux et intress par la vie	50,9 (40,6 61,1)	58,4 (56,5 60,3)	64,4 (63,2 65,7)
Plutt heureux	42,1 (31,9 52,4)	33,8 (31,9 35,7)	29,9 (28,7 31,1)
Plutt malheureux/ malheureux et peu intress la vie/si malheureux que la vie ne vaut pas la peine d'tre vcue	7,0 (2,7 11,3)^ E^	7,8 (6,7 8,9)	5,6 (5,1 6,2)
Rpercussions de la prestation de soins^ g^			
Se sent inquiet ou anxieux^ f^			
Oui	70,1 (59,0 81,2)	62,5 (60,3 64,7)	S.O.
Non	29,9 (18,8 41,0)^ E^	37,5 (35,3 39,7)	S.O.
Se sent fatigu			
Oui	68,0 (57,3 78,6)	59,4 (57,2 61,7)	S.O.
Non	32,0 (21,4 42,7)^ E^	40,6 (38,3 42,8)	S.O.
Se sent dbord^ f^			
Oui	57,8 (46,8 68,8)	42,5 (40,3 44,7)	S.O.
Non	42,2 (31,2 53,2)	57,5 (55,3 59,7)	S.O.
A des problmes de sommeil^ f^			
Oui	48,6 (37,9 59,2)	41,1 (38,8 43,3)	S.O.
Non	51,4 (40,8 62,1)	58,9 (56,7 61,2)	S.O.
Se sent colrique ou irritable^ f^			
Oui	45,3 (34,8 55,8)	42,7 (40,5 44,9)	S.O.
Non	54,7 (44,2 65,2)	57,3 (55,1 59,5)	S.O.
Se sent dprim^ f^			
Oui	28,7 (20,2 37,3)	26,1 (24,3 28,0)	S.O.
Non	71,3 (62,7 79,8)	73,9 (72,0 75,7)	S.O.
Se sent seul ou isol^ f^			
Oui	24,5 (16,3 32,7)^ E^	24,3 (22,5 26,2)	S.O.
Non	75,5 (67,3 83,7)	75,7 (73,8 77,5)	S.O.
Perd l’apptit^ f^			
Oui	15,6 (8,9 22,3)^ E^	13,8 (12,4 15,2)	S.O.
Non	84,4 (77,7 91,1)	86,2 (84,8 87,6)	S.O.
prouve du ressentiment^ f^			
Oui	15,1 (8,6 21,6)^ E^	25,2 (23,5 27,0)	S.O.
Non	84,9 (78,4 91,4)	74,8 (73,0 76,5)	S.O.
Mesure dans laquelle les expriences de prestation de soins ont t gratifiantes^ f^			
Trs gratifiantes/gratifiantes	68,3 (58,5 78,1)	54,2 (52,0 56,5)	S.O.
Un peu ou pas du tout gratifiantes	31,7 (21,9 41,5)	45,8 (43,5 48,0)	S.O.
Passe moins de temps se dtendre ou prendre soin de soi-mme en raison de son rle d’aidant			
Oui	67,0 (56,7 77,3)	58,7 (56,4 61,0)	S.O.
Non	33,0 (22,7 43,3)	41,3 (39,0 43,6)	S.O.
Mesures de soutien et besoins de soutien non satisfaits			
Le bnficiaire de soins a galement reu l’aide de professionnels^ h^			
Oui	73,9 (64,0 83,7)	60,0 (58,0 62,1)	S.O.
Non ou ne sait pas	26,1 (16,3 36,0)^ E^	40,0 (37,9 42,0)	S.O.
L’aidant a reu un soutien dans ses tches d’aidant^ i^			
Oui	87,4 (81,3 93,6)	70,7 (69,1 72,2)	S.O.
Non	12,6 (6,4 18,7)^ E^	29,3 (27,8 30,9)	S.O.
Besoins de soutien non satisfaits^ j^			
Oui	46,8 (37,0 56,6)	29,6 (27,8 31,5)	S.O.
Non	53,2 (43,4 63,0)	70,4 (68,5 72,2)	S.O.

**Source**: 2018 General Social Survey – Caregiving and Care Receiving.^12^

**Abbreviations**: CI, confidence interval; DD, developmental disabilities or disorders; NA, not applicable. 

**Notes**: Percentages and 95% CIs are based on weighted data. The 95% CI shows an estimated range of values that is likely to include the true value 19 times out of 20. 

^a^ “DD caregivers” are caregivers of individuals with developmental disabilities or disorders. Unweighted n = 248; weighted n = 333 869. 

^b^ “Other caregivers” are caregivers of individuals with conditions other than developmental disabilities or disorders. Unweighted n = 7416; weighted n = 7 438 728. 

^c^ “Non-caregivers” are individuals who were not caregivers. Unweighted n = 12 594; weighted n = 22 982 588. 

^d^ The *Employment Equity Act* defines members of visible minorities as “persons, other than Aboriginal peoples, who are non-Caucasian in race or non-white in colour.”^13^ Accordingly, population groups were categorized as “visible minorities” or “not visible minorities.” “Not visible minority” includes those who identified as White, Indigenous, and multiple origin White/Latin American and White/Arab-West Asian.^14^ The “visible minority” group includes South Asian, Chinese, Black, Filipino, Latin American, Arab, Southeast Asian, West Asian, Korean, Japanese, and other.^14^

e Includes spouse/partner, ex-spouse/ex-partner, father, mother, grandfather, grandmother, son-in-law, daughter-in-law, father-in-law, mother-in-law, brother-in-law, sister-in-law, nephew, niece, uncle, aunt, cousin, close friend, neighbour, co-worker and other. 

f Only asked of respondents who were interviewed by non-proxy.

g Asked of caregivers who provided at least 2 hours of care per week. 

h Only asked of caregivers with care receivers who did not live in an institution. 

i Included if caregivers received support in any of the following ways: spouse or partner modified their life or work arrangements; children, extended family members, close friends or neighbours, or community, spiritual community or cultural or ethnic groups provided help; occasional relief or respite care; family or friends provided financial support, received money from government programs, or received federal tax credits. 

j Included if caregivers responded “yes” to the following question: “Is there any other type of support that you would like to have to help with your caregiving duties?” 

E Use with caution.

F Too unreliable to be published due to high sampling variability (coefficient of variation > 33.3%). 

The mean age of the care receivers with DD was 22.5 years (Table 1). Almost two-thirds were male (64.2%) and the children of the caregivers (62.2%) versus another relationship. More than three-quarters lived in the same household as the caregiver (79.2%) and had at least one other caregiver, paid or unpaid (83.9%). Compared with care receivers with other conditions, those with DD were significantly younger and significantly more likely to be male, children of the caregiver, living in the same household as the caregiver and have at least one other caregiver.

***Care provided***

DD caregivers provided an average of 29hours of care per week (Table 1), most commonly help with transportation (86.2%); meal preparation, meal clean-up, house cleaning, laundry or sewing (80.0%); and scheduling or coordinating care-related tasks (60.5%). Compared with other caregivers, DD caregivers provided significantly more hours of care per week (29.1 vs. 13.4 hours) and were significantly more likely to provide each type of care.

***Health status***

Less than half of DD caregivers described their general health and mental health as excellent or very good (40.5% and 45.7%, respectively), and only one-quarter reported that most days were not at all or not very stressful (Table 1). However, more than three-quarters reported that they were satisfied or very satisfied with life (77.2%), and about half indicated that they were happy and interested in life (50.9%). Compared with non-caregivers, DD caregivers reported less optimal general and mental health, more stress, and less life satisfaction and happiness.

***Impacts of caregiving***

DD caregivers most commonly described feeling worried or anxious as a result of their caregiving responsibilities (70.1%); having rewarding or very rewarding caregiving experiences (68.3%); feeling tired as a result of their caregiving duties (68.0%); and spending less time relaxing or taking care of themselves due to their caregiving (67.0%) (Table 1). While other caregivers experienced similar impacts, a significantly higher proportion of DD caregivers reported feeling overwhelmed (57.8% vs. 42.5%). Conversely, a significantly smaller proportion of DD caregivers reported feeling resentful (15.1% vs. 25.2% for other caregivers), and a significantly higher proportion found caregiving to be very rewarding or rewarding (68.3% vs. 54.2% for other caregivers).

***Supports and unmet support needs***

**Figure 1:**
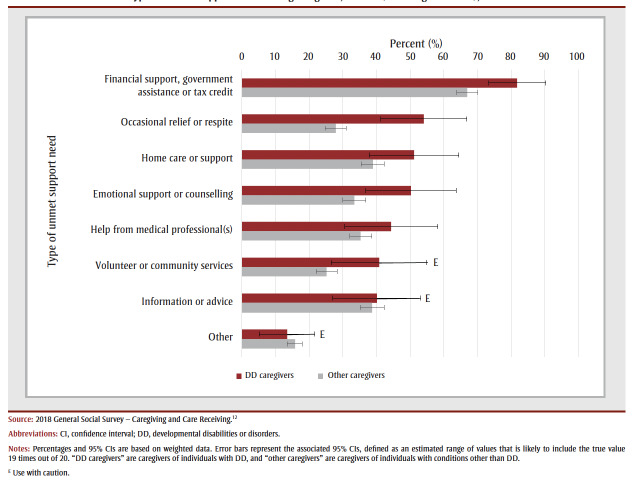
Types of unmet support needs among caregivers, Canada (excluding territories), 2018

Almost three-quarters (73.9%) of care receivers with DD who did not live in an institution also received help from professionals (i.e. paid workers or organizations), and most (87.4%) DD caregivers received some type of support to accommodate their caregiving duties (Table 1). Despite this, almost half (46.8%) of DD caregivers reported unmet support needs, with the most common being financial support, government assistance or tax credit, occasional relief or respite care, home care or support, and emotional support or counselling (Figure 1) . Compared with other caregivers, a significantly higher proportion of DD caregivers received help from professionals and some type of support in their caregiving duties, yet still had unmet support needs (Table 1).

## Discussion

This is the first study to use data from the GSS to report on the characteristics of DD caregivers in Canada and the impacts of their caregiving experiences. We found the proportion of DD caregivers with optimal health to be smaller than the proportion of non-caregivers with optimal health; more than two-thirds of DD caregivers felt worried or anxious and felt tired as a result of their caregiving roles. Almost half also reported various unmet support needs, with financial support, occasional relief or respite care, and home care or support the most common. Despite these challenges, many DD caregivers found their caregiving experiences rewarding.

Previous Canadian and international studies have also shown that caregiving for individuals with DD can negatively affect caregivers’ mental and physical health, with effects varying with the caregiver, care receiver, family characteristics and circumstances such as caregiver income, the care receiver’s age and the number of care receivers with disabilities being looked after by each caregiver, and barriers to accessing services and supports.^5^,^9^,^10^,^11^,^15^,^16^ We found that DD caregivers were more likely than other caregivers to care for their own children and for younger individuals, to live in the same household as the care recipient and to have the support of at least one additional caregiver. DD caregivers also provided more hours of care per week than other caregivers. These differences in demographics and circumstances may have important implications on the experience and impacts of caregiving.^15^ However, exploring the specific factors associated with the effects of caregiving was beyond the scope of this study.

Prior investigations have identified unmet support needs for both individuals with DD and their caregivers, with inadequate support partly explaining why families are often negatively affected as a result of their children’s disabilities.^17^,^18^ A recent survey of caregivers found the most frequently reported support needs to be related to mental health and finances, with variations across sociodemographic groups.1 Our study also found that financial support was the most commonly reported unmet need, followed by occasional relief or respite care, home care or support, as well as emotional support or counselling. Financial credits^19^-^21^ and organizations that offer education and training, peer support, advocacy and counselling opportunities for caregivers are available in Canada, although eligibility and availability vary depending on the caregiver’s situation.

Although research often focuses on the burdens of caregiving, previous studies have shown that parents of children with DD report positive aspects of their caregiving experiences, seeing their child as a source of happiness, personal strength and growth, and family closeness.^22^,^23^ These perceptions, which mirror our findings of more rewarding caregiving experiences among DD caregivers, have been linked to caregivers’ healthy coping strategies and access to caregiving supports.^23^

***Strengths and limitations***

This study used data from the 2018 GSS, a large, population-based survey with weighted estimates representative of the target population; however, some limitations are worth noting. First, the survey did not include the territories, limiting the generalizability of the findings. Second, the study sample size prevented us from disaggregating some sociodemographic characteristics such as ethnicity and Indigeneity. Third, the survey did not capture specific types of DD; it was therefore not possible to examine the differential impacts of specific DD on caregivers’ experiences. Lastly, while non-overlapping CIs indicate significant differences, overlapping CIs do not necessarily imply a lack of difference. However, the use of this conservative approach minimizes drawing erroneous conclusions of significance. Further, the small sample of DD caregivers resulted in wider CIs, potentially limiting our ability to detect significant differences.

The findings from this study reflect the experiences of DD caregivers prior to the COVID-19 pandemic. The pandemic, particularly early on, frequently exacerbated caregiving challenges through disruptions to routines, education, services or supports.^24^-^28^

## Conclusion

Despite the negative impacts of caregiving, such as worse general and mental health, a higher proportion of DD caregivers than of other caregivers described their caregiving experiences as rewarding. Still, a large proportion of DD caregivers had unmet support needs. These findings underscore the importance of supports and services for DD caregivers to manage the challenges and enhance the positive aspects of caregiving. 

Future cycles of the GSS will allow for monitoring of the burden of caregiving on this population over time. Additional research could examine the varied impacts on caregivers of individuals with different types of DD and explore differences in experiences by caregiver, care receiver and family demographics and circumstances.

## Funding

None.

## Conflicts of interest

The authors have no conflicts of interest.

## Authors’ contributions and statement

SP: Conceptualization, formal analysis, writing – original draft, writing – review and editing.

SO: Conceptualization, writing – original draft, writing – review and editing. 

SS: Visualization, writing – original draft, writing – review and editing. 

All authors approved the final version of this manuscript.

The content and views expressed in this article are those of the authors and do not necessarily reflect those of the Government of Canada.
